# “Sigmoid diverticulitis mimicking cholecystitis” a clinical challenge

**DOI:** 10.1186/s13089-019-0127-6

**Published:** 2019-06-11

**Authors:** Ilaria Pulzato, Enrico Boero, Elona Shaipi, Luciano Cardinale

**Affiliations:** 1 Dipartimento di Medicina interna e Specialità mediche, Di.M.I. Università degli Studi di Geniva, Viale Benedetto XV, 10, 16132 Genoa, GE Italy; 2 Dipartimento di scienze Chirurgiche, Università degli Studi di Torino, Turin, Italy; 3 SCDU Radiodiagnostica e Medicina Nucleare, Azienda Ospedaliero-Universitaria S.Luigi Gonzaga, Regione Gonzole 10, 10043 Orbassano, TO Italy

**Keywords:** Diverticulitis, Ultrasound, Computed Tomography, Colon embryologic abnormalities

## Abstract

Diverticular disease is a common disorder and its incidence increases with ageing. Pathophysiology is multifactorial. Lifestyle, including smoking, alcohol intake, decreased dietary fibres and lack of physical activity, plays a predominant role. Genetics seems also to contribute specifically for right-sided diverticular disease (RSD). The majority of the patients with diverticular disease are asymptomatic. Diverticulitis is the inflammation of the diverticula usually presenting with abdominal pain associated to nausea, vomiting, rectal bleeding, diarrhoea and fever. When the inflammation process affects the diverticula in the ascending colon, the condition represents a clinical challenge as it can be easily misdiagnosed with other acute abdominal emergencies. We reported a case of a 70-year-old female who presented to our Emergency Department (ED) with right upper quadrant pain and an initial clinical suspicion of cholecystitis. Ultrasound (US) and Computed Tomography (CT) demonstrated an anatomical variation of the sigmoid colon diverticulitis. This clinical report demonstrates that ultrasound plays a relevant part as first-step approach to the acute abdominal conditions and its accuracy increases together with other diagnostic tools such as Computer Tomography.

## Background

Diverticular disease is a common disorder affecting almost 50% of the population over 60 years. Its incidence increases with ageing, reaching up to 80% in the population older than 80 years [1]. Diverticula consist of outpouching from the large intestine wall, occurring mainly in the sigmoid colon. Pathophysiology of diverticulosis is not yet completely understood. Many factors such as colonic hypermobility, micro oral content and visceral hypersensitivity are involved in its development. Lifestyle, including smoking and alcohol intake, decreased dietary fibres and lack of physical activity, plays a predominant role [2]. Genetics seems to be involved specifically in the right-sided diverticular disease (RSD), which has an incidence of approximately 8.5% in the Western and 40% in the Eastern population [3]. Up to 90% of the patients with diverticular disease are asymptomatic. Few of them can develop large intestine carcinoma, whereas solitary diverticulum of the caecum may occur as a congenital lesion [3].

Diverticulitis is the inflammation of the diverticula, which can lead to complications such as abscess or perforation. Presenting symptoms are usually pain, nausea, vomiting, rectal bleeding, diarrhoea and fever. When the inflammation process affects the diverticula in the right-sided large intestine, it becomes a clinical challenge, as it can be easily misdiagnosed with other acute abdominal emergencies, such as appendicitis, cholecystitis, appendagitis [4].

The distinction between these acute abdominal conditions is relevant for the prognosis, as RSD is treated conservatively, and abscess formation is rare.

## Case presentation

A 70-year-old female presented to our ED with right upper quadrant abdominal pain of 1-day duration described as sharp in nature, which started suddenly and became constant over time. No nausea, vomiting, melena, hematochezia or hematemesis were reported. The patient’s past medical history was irrelevant; she also had no family history of inflammatory bowel disease or gastrointestinal malignancies. Physical examination was unremarkable, apart from right superior quadrant tenderness with localised guarding. Vital parameters were within the normal range. Blood tests results showed elevated white blood cell count (14,500 per microlitre with 79.2% neutrophils), while electrolytes and pancreas function were within the limits.

### Management and outcome

**Fig. 1:**
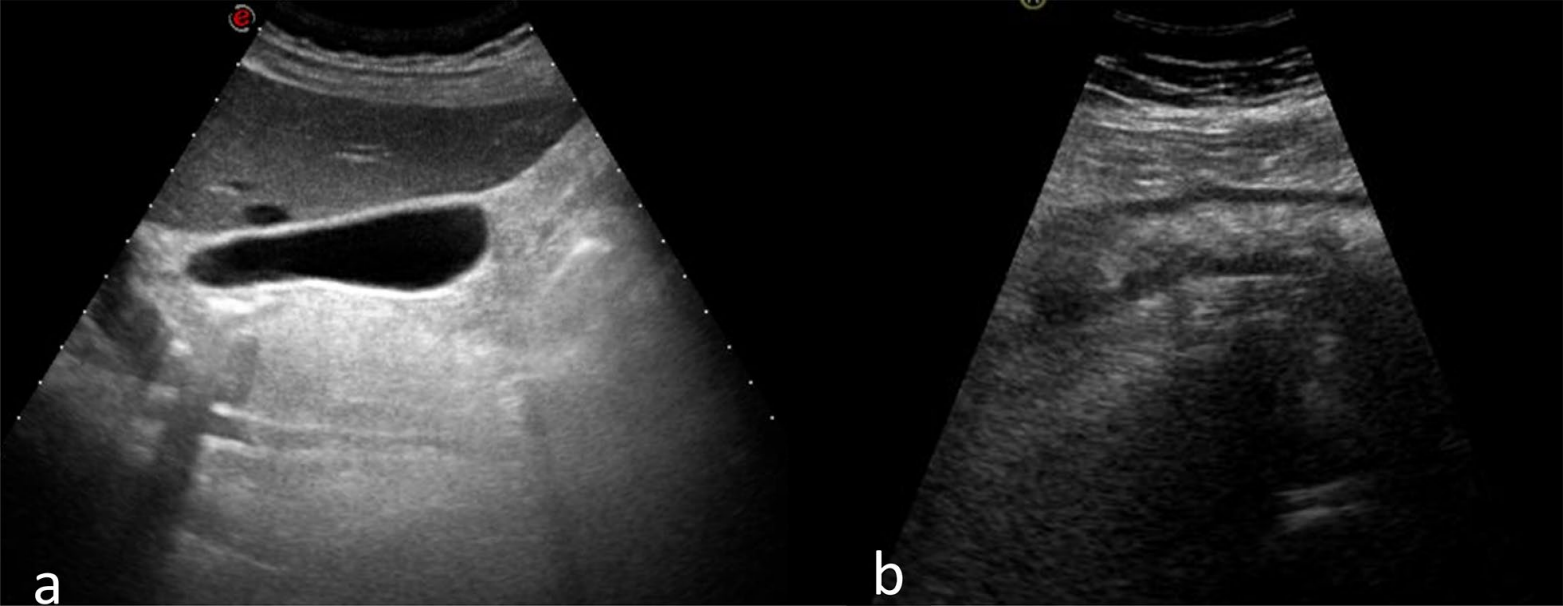
The ultrasound demonstrated normal liver, spleen, kidneys, no free fluid and normal gall bladder (**a**). A diffuse wall thickening of the bowel in the right upper quadrant (**b**). The impression was of a right-sided inflammatory bowel disease or colon diverticulitis

**Fig. 2:**
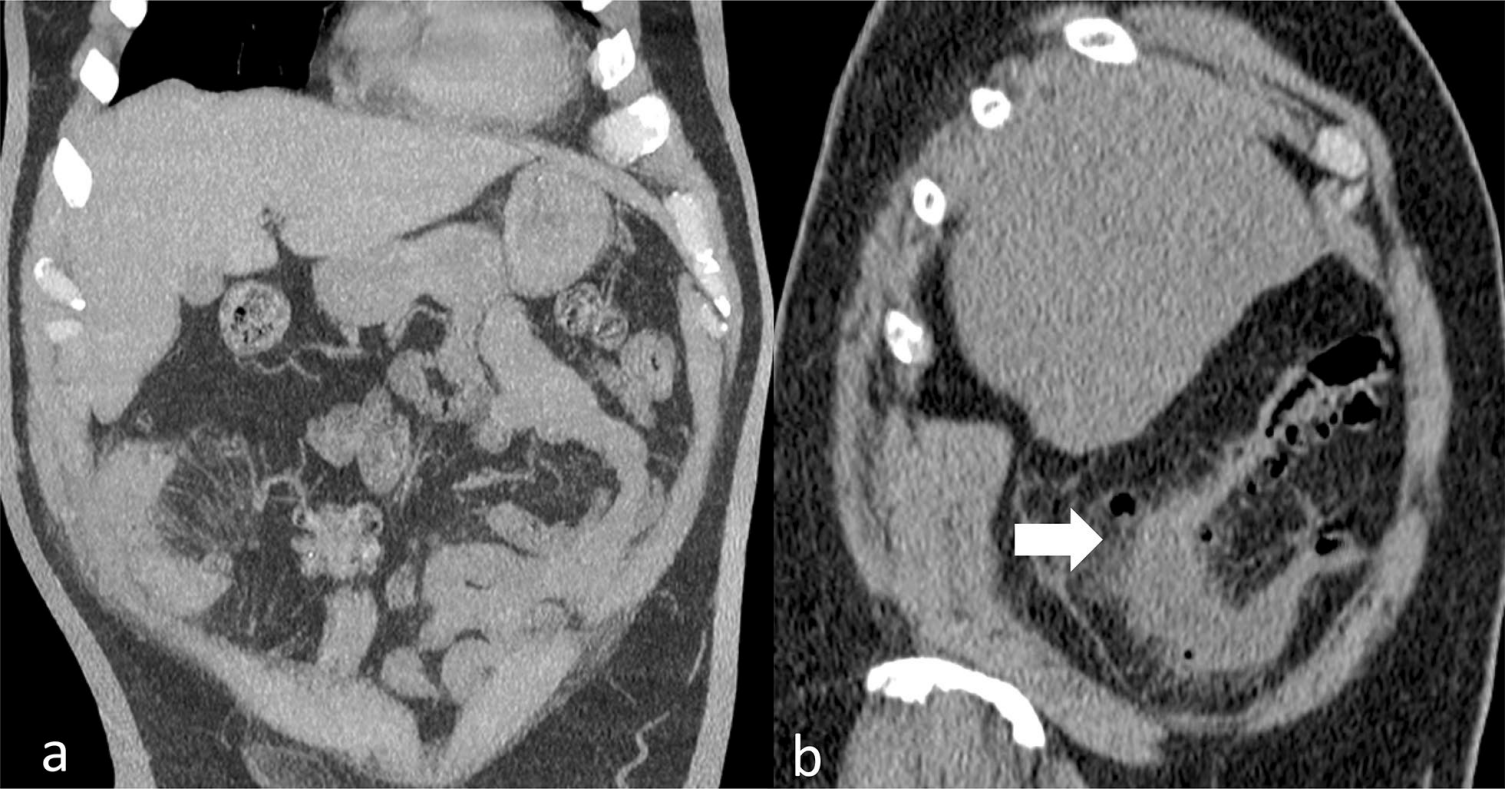
The CT scan of the abdomen and pelvis without intravenous contrast material. In **a**, the coronal reformat and in **b** the sagittal reformat. The scan showed the wall thickening of the left and sigmoid colon, with diverticular outpouching. Moreover, there was stranding of the perivisceral fat due to perivisceral tissue inflammation complicated with limited perforation and a small free air pocket next it; therefore, the diagnosis favoured was diverticulitis with limited perforation (white arrow). There was no free fluid

In the broad spectrum of differential diagnosis, gallbladder disease, appendicitis, peptic disease and pancreatitis were considered. According to symptom presentation and hospital policy, an abdominal ultrasound was initially performed. This showed normal appearance of the abdominal solid structures (Fig. 1a), apart from a marked segmental large bowel-wall thickening on the right hypochondrium surrounded by hyperechoic, non-compressible fatty tissue and a small fluid collection. According to these findings, the clinical suspicion was either inflammatory bowel disease or diverticulitis. The patient underwent a Computed Tomography (CT) which confirmed wall thickening of the descending and sigmoid colon, diverticular outpouching and stranding of perivisceral fat complicated with localised perforation. Due to the patient’s inborn anatomical variant of redundancies of large intestine, also known as dolichocolon condition, her sigmoid colon was displaced on the right-side of the abdomen (Fig. 2a, b). The patient was referred to the surgical team and admitted to the ward for intravenous fluid therapy and antibiotics. She was discharged after a week with clinical follow-up.

## Discussion

Diverticula are defined as sac-like protrusions from the large intestine wall due to a combination of anatomical, dietary, motility and structural influences [5]. They are divided in two general anatomical types, true and false. The majority of the colon diverticula are false diverticula (also known as pseudodiverticula), composed of mucosa and submucosa protruding through the muscularis externa, covered only by serosa. They are acquired, multiple and frequently located in the sigmoid colon. True diverticula involve all three layers of the bowel wall (mucosa, submucosa and muscularis externa) as seen in most congenital diverticula such as Meckel’s diverticula and in the diverticula of right-sided large intestine [6, 7]. They are mostly congenital and solitary [8].

When infection of the diverticulum occurs, it can manifest with a spectrum of symptoms such as pain, fever, diarrhoea, nausea, vomiting and bloody stools. Right-sided diverticulitis (RSD) may mimic several abdominal emergencies such as cholecystitis, appendicitis or epiploic appendagitis. The distinction between these conditions and RSD is crucial as the latter is treated conservatively and abscess formation is rare [9, 10]. Within the range of possible investigations, US plays its role being the first imaging study modality performed in patients presenting with abdominal pain. It can safely rule out cholecystitis and identify several abdominal conditions such as epiploic appendagitis (EA), appendicitis, colitis, neoplastic lesion and Crohn’s disease, each of them characterised by specific sonographic aspects.

The sonographic appearance of the inflamed diverticulum is defined as hypoechoic wall thickening, due to oedema and muscular hypertrophy. US can also reveal complications such as inflammation of the pericolic fat and free fluid collection around the target area. Specifically, the pericolic fat stranding consists of hyperechoic non-compressible mass-like finding whether the free fluid has a hypoechoic ultrasound aspect [11]. Give US its crucial role in the decision-making process, the CT remains the modality of choice in the diagnosis of diverticulitis, due to its ability to identity the anatomical location along the large intestine, the involvement of adjacent organs and the presence of complications such as perforation, abscess and fistula [7]. In summary, ultrasound evaluation of abdominal emergencies is a rapid and useful first-level investigation that may lead the next step in the clinical decision-making process, such as a second-level imaging study, surgical or conservative approach. Both imaging modalities have their advantages and limitations, making their combined use crucial for the best appropriate clinical management of patients.

## Data Availability

Not applicable.

## Abbreviations

ED: Emergency Department
CT: Computed Tomography
RSD: right-sided diverticulitis
US: ultrasound
EA: epiploic appendagitis
